# Helminth Egg Automatic Detector (HEAD): Improvements in development for digital identification and quantification of helminth eggs and their application online

**DOI:** 10.1016/j.exppara.2020.107959

**Published:** 2020-10

**Authors:** B. Jiménez, C. Maya, G. Velásquez, J.A. Barrios, M. Perez, A. Román

**Affiliations:** Instituto de Ingeniería, UNAM, P.O. Box 70-186, México, D.F., 04510, Mexico

**Keywords:** Automatic identification, Helminth eggs, Helminthiasis, Microscopy image analysis, Wastewater

## Abstract

Helminths are parasitic worms that constitute a major public health problem. Conventional analytical techniques to evaluate helminth eggs in environmental samples rely on different steps, namely sedimentation, filtration, centrifugation, and flotation, to separate the eggs from a variety of particles and concentrate them in a pellet for direct observation under an optical microscope. To improve this process, a new approach was implemented in which various image processing algorithms were developed and implemented by a Helminth Egg Automatic Detector (HEAD). This allowed identification and quantification of pathogenic helminth eggs of global medical importance and it was found to be useful for relatively clean wastewater samples. After the initial version, two improvements were developed: first, a texture verification process that reduced the number of false positive results; and second, the establishment of the optimal thresholds (morphology and texture) for each helminth egg species. This second implementation, which was found to improve on the results of the former, was developed with the objective of using free software as a platform for the system. This does not require the purchase of a license, unlike the previous version that required a Mathworks® license to run. After an internal statistical verification of the system was carried out, trials in internationally recognized microbiology laboratories were performed with the aim of reinforcing software training and developing a web-based system able to receive images and perform the analysis throughout a web service. Once completed, these improvements represented a useful and cheap tool that could be used by environmental monitoring facilities and laboratories throughout the world; this tool is capable of identifying and quantifying different species of helminth eggs in otherwise difficult environmental samples: wastewater, soil, biosolids, excreta, and sludge, with a sensitivity and specificity for the TensorFlow (TF) model in the web service values of 96.82% and 97.96% respectively. Additionally, in the case of *Ascaris*, it may even differentiate between fertile and non-fertile eggs.

## Highlights

• It provides uniform criteria for helminth egg identification, which reduces process uncertainty.
• It confers better species classification, due to morphologic and texture characteristics.
• It results in a reduction in the time required for identification and quantification.
• The software will be available as a cloud service.
• It is expected that further steps in the software's development will increase its current capabilities and potential.

## Graphical abstract

**Figure:**
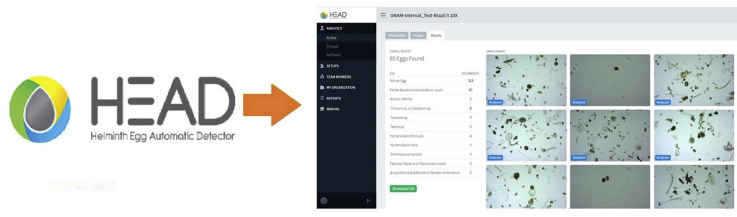

## 1 Introduction

Helminths are the source of health risks associated with poor sanitation, the use of contaminated water for irrigation and the disposal of excreta or sludge to soil. They are transmitted via their eggs and/or larvae, which often begin their development cycle within the human host. They enter the host when they are actively ingested or penetrate the skin. In rare cases they may also be inhaled. Due to their low infectious dose, their high persistence in the environment and their high resistance to conventional treatment processes, helminth eggs are considered to be the biological structures most resistant to inactivation in the field of environmental engineering (WHO, 2006; Jiménez et al., 2016).

With regard to their health implications, helminths cause a set of diseases called “helminthiasis” that are specifically named for the genera involved (for instance Ascariasis for *Ascaris*). Symptoms include deterioration of the intestines, toxic effects, blood loss, diarrhea, undernourishment, and anemia. Some helminths eat away at the intestinal wall causing hemorrhages while, simultaneously, they secrete compounds that prevents blood coagulation. The damage caused by inflammation and the channels they open result in tumors and excrescences. In addition, helminths can block conduits (for example, the biliary ducts) or cause intestinal obstruction and perforation of the digestive tract, giving rise to peritonitis (WHO, 2006; Bogitsh et al., 2013). Helminthiasis constitutes a major public health problem, particularly in developing countries. Helminthiasis are endemic in Africa, Latin America, and East Asia. It is estimated that around half of the global population is infected, in particular children between 5 and 15 years of age (UN, 2003; Steinmann et al., 2006; Mascarini-Serra, 2011; Lustigman et al., 2012; WHO, 2012a, 2012b; Pullan et al., 2014; Strunz, 2014; Jiménez et al., 2016).

Global, international, and local regulations often establish the limit of helminth eggs in wastewater for irrigation and sludge for application in agriculture, representing a concentration that does not pose a health risk (NOM-001-SEMARNAT-1999; NOM-004-SEMARNAT-2002; USEPA, 1992; WHO, 1989; WHO, 2006). These limits are less than one egg per liter for water (<10 E/L) and less than one egg per gram of dry sludge (≤1 H E/gTS) (WHO, 2006). However, traditional methods to quantify and ensure compliance with such regulations involve, as a final step, identification through direct observation under the optical microscope. For this, highly qualified personnel and considerable amounts of time are required, sometimes becoming a source of error during quantification (Maya et al., 2006).

Another common problem is that there is also a great number of objects (artifacts) with similar size, shape, and even color that may appear in the images and could be mistakenly identified as helminth eggs. This is especially true in samples with a high content of debris such as sludge, soils, and excreta. Some examples of these different confounding objects include vegetable waste, salt crystals, medical substances, proteins, digesting deficiency, starches by cell debris, fat by cooling feces, yeast, pollen, or pine spores (Blanco and Galiano, 1989).

To solve this problem, in 2016 a new processing system for automatic identification and quantification of helminth eggs (Helminth Eggs Automatic Detector, HEAD) was developed by Jiménez et al. (2016), based on images taken under a microscope. This automatic system reduces both the potential for subjective error and the time required for visual enumeration under the optical microscope; in addition, it is able to efficiently identify and quantify helminth eggs commonly found in wastewater samples with consistent results i.e. *Ascaris* spp*.* (*lumbricoides* and *suum*) *-*fertile and infertile eggs-, *Toxocara canis*, *Trichuris trichiura*, *Taenia* spp*. (solium and saginata)*, *Hymenolepis diminuta*, and *Schistosoma mansoni*. The main advantage of using this system is that it does not require highly trained personnel (i.e. expert parasitologists). Its application also confers the following advantages: 1) it provides uniform criteria for the identification of helminth eggs, which reduces the uncertainty of the process; 2) it allows better classification of the species, due to their morphological and textural characteristics, and 3) it reduces the time required for identification and quantification. The flexibility of the image processing tools allows an increase in their identification capabilities in terms of the type of sample and the species that can be included in the identification database.

Ongoing work with HEAD has allowed continuous improvements to be made. These include the design and implementation of new algorithms, a stage of selecting the appropriate characteristics of the various species of helminth eggs that will be detected and classified, and an increase in the number of environmental samples able to be processed. The latter allows the system to be applied not only to clean wastewater (as was the case for the first version of the system), but, also to wastewater with a high content of debris, sludge, biosolids, soils, and excreta. In addition, the system is capable of configuring a reference database of digital images to calibrate software in C # with. NET framework. This is based on the previously-tested methodology and assigned to a remote server to establish a server-client web service model. In this way, it is possible to receive and process images automatically, as well as to directly report the results without the need for specific operators. This is possible due to the incorporation of different image processing tools and pattern recognition algorithms (Perona and Malik, 1990; Belanche-Muñoz and Blanch, 2008; Dogantekin et al., 2008; Avci and Varol, 2009).

## 2 Materials and methods

### 2.1 Improvements and development of the system for digital identification

#### 2.1.1 Selection of helminth eggs species

**Fig. 1:**
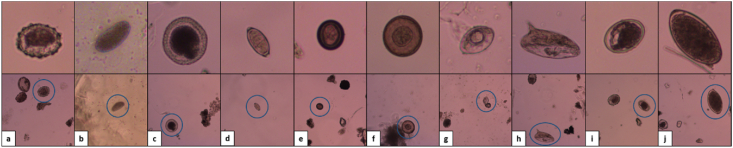
Isolated (top) and whole microscopic images (bottom) of the selected helminth egg species: (a) fertile *Ascaris lumbricoides*, (b) Infertile *Ascaris lumbricoides*, (c) *Toxocara canis* (d) *Trichuris trichiura,* (e) *Taenia* spp*.,* (f) *Hymenolepis diminuta,* (g) *Hymenolepis nana, (*h) *Schistosoma mansoni,* (i) *Hookworm (Ancylostoma duodenale* and *Necator amerianus),* and (j) Flukes (*Fasciola hepatica* and *Fasciolopsis buski*).

As reported by Jiménez et al. (2016) the system was capable of identifying six species of helminth eggs with adequate sensitivity (86%) in clean wastewater samples. These species were selected based on their medical importance and global ubiquity: *Ascaris* spp*. (lumbricoides* and *suum)* -fertile and infertile eggs-, *Toxocara canis*, *Trichuris trichiura, Taenia* spp*. (solium* and *saginata*)*, Hymenolepis diminuta,* and *Schistosoma mansoni.* In this first stage of development *Hymenolepis nana* was also included; however, due to its transparency and small size (35 μm) its proper identification was difficult, even for trained technicians, so it was not considered further. In the present work, the number of species was increased to include: *Hymenolepis nana,* Hookworms (*Ancylostoma duodenale*, *Necator americanus),* Flukes *(Fasciola hepatica,* and *Fasciolopsis buski)* (Fig. 1).

Images were taken using a Carl Zeiss AxioLab A1 optical microscope and an Imaging Development Systems UI-1480LE-C-HQ USB2 color camera. To collect homogenous images, all photographs were acquired using a 10× objective and 2560 × 1920 pixel resolution without compression indicated in the color camera software.

### 2.2 Segmentation and kind of environmental sample

**Fig. 2:**
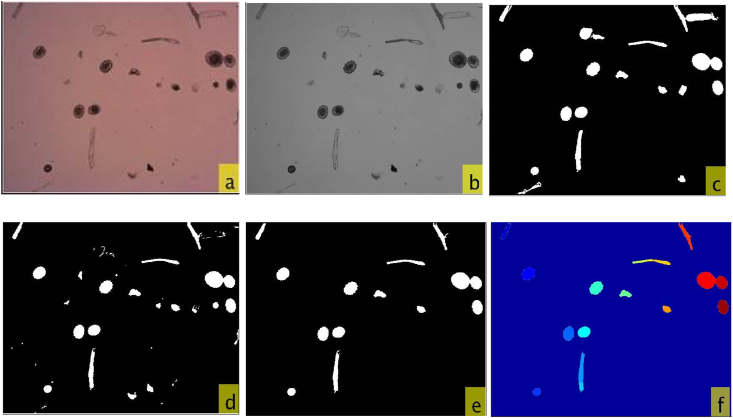
(2a) Original image, (2b) image after the grayscale conversion, (2c) and (2d) result of the binarizations, (2e) final segmentation, and (2f) image after watershed filter.

As mentioned previously, the system was adapted for application not only to clean wastewater, but also to sludge, biosolids, soils, and excreta samples. Certain variations in the segmentation algorithm of the system are necessary depending on the type of sample it will analyze. This requires user input before running an analysis in order to perform the best sequence of processes and filters on each image. These variations were programmed into the system due to the nature of certain artifacts and slight variations on the eggs’ characteristics, improving results significantly in images with a large amount of debris. The combination of edge detection with a local threshold algorithm provided good results (Fig. 2).

As shown in Fig. 2, the first step was the acquisition of the original color image (2a). This was transformed to a gray level image (2b) and subsequently filtered by the Anisotropic Diffusion Filter to reduce noise and preserve the image edges (Perona and Malik, 1990). Following this, edge detection binarization of the image (2c) was performed using a threshold of a Laplacian of Gaussian (*LoG*) filter. The Laplacian is a 2-D isotropic measure of the second spatial derivative of an image. The Laplacian of an image highlights the regions of rapid intensity change and is, therefore, often used for edge detection. In addition, it is often applied to an image that has first been smoothed with a Gaussian filter in order to reduce its sensitivity to noise. With the Gaussian standard deviation σand being *x* and *y* the coordinates of a pixel, the *LoG* function centered on cero has the following form:

(1) $$LoG\left(x,y\right)=-\frac{1}{\pi \sigma^{4}}\;\left[1-\frac{x^{2}+y^{2}}{2\sigma^{2}}\right]e^{-\frac{x^{2}+y^{2}}{2\sigma^{2}}}$$

where:The pixel coordinates are (x,y) and σ is the standard deviation over a predefined window (25 pixels length in the case of the current study based on the size of the egg borders for images with a size of 2560 × 1920 pixels).

After anisotropic filtering, the local threshold algorithm (Sauvola and Pietikainen, 2000) was applied (2 d) to detect the image regions with possible eggs. This method considers that if the image presents a significant amount of local contrast, as is the case for most of the acquired microscopy images, the threshold should be chosen close to the mean value, whereas if there is little contrast, the threshold should be chosen below the mean, by an amount proportional to the normalized local standard deviation. In Sauvola's binarization method, the threshold t(x,y) is computed using the mean m(x,y) and standard deviation s(x,y) of the pixel intensities in a m×m window centered on the pixel (x,y):

(2) $$t\left(x,y\right)=m\left(x,y\right)\left[1+k\left(\frac{s\left(x,y\right)}{R}-1\right)\right]$$

where:

*R* is the maximum value of the standard deviation (R=128for a grayscale image).

k is a parameter which takes positive values in the range [0.2,0.5].

Local mean m(x,y).

Standard deviation s(x,y) adapts the value of the threshold according to the contrast in the local neighborhood of the pixel.

The final segmentation was carried out by considering only the common pixels between the *LoG* and the Sauvola binarizations (2e). In doing so it is ensured that the possible egg region will be only the one that could possibly belong to a real egg.

**Fig. 3:**
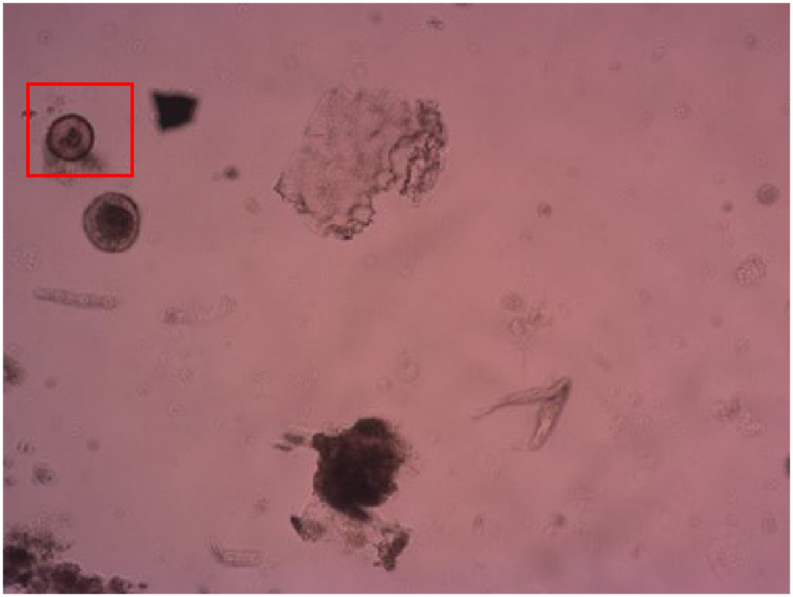
Original image from a sludge sample, with a *Hymenolepis diminuta* egg.

**Fig. 4:**
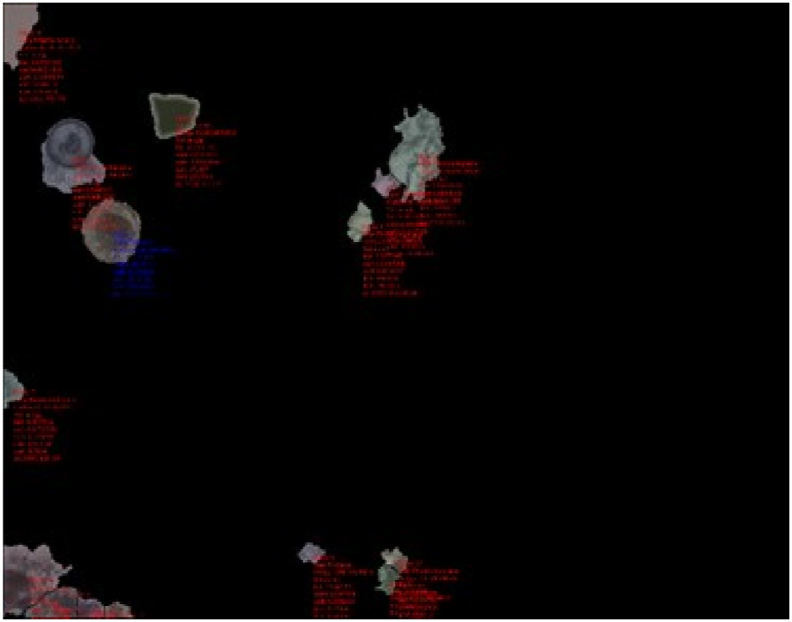
HEAD result with only the LoG filter (*H. diminuta* egg detected).

This methodology is especially useful in cases where the images are from sludge and excreta samples, and where the eggs are commonly found in contact with debris, whose characteristics make it difficult for the *LoG* algorithm to separate the possible egg in the analysis; for example, the *Hymenolepis diminuta* egg, in the left corner of the image, where the debris modify the egg border (Fig. 3) is not detected by the *LOG* algorithm (Fig. 4). The local threshold filter helps to improve segmentation in objects that have transparent debris stuck to their borders.

**Fig. 5:**
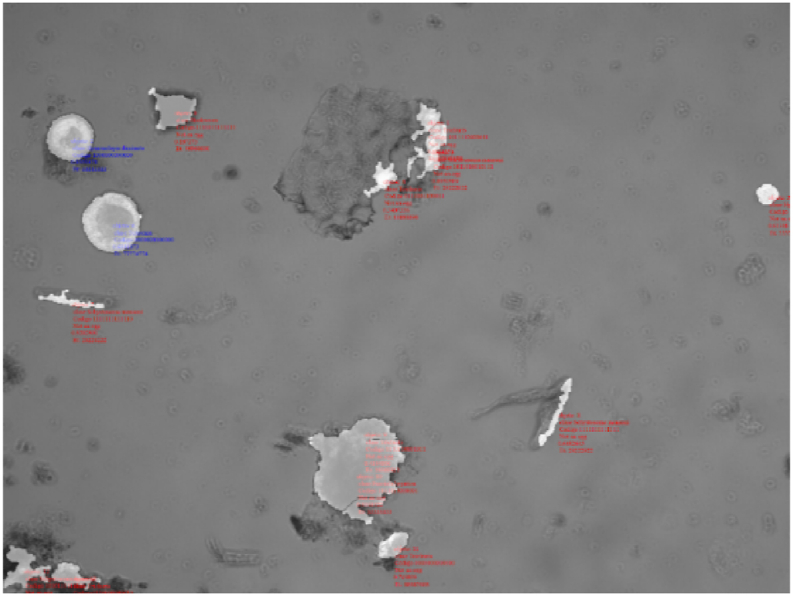
HEAD result with the union of the LoG and Sauvola filters.

Using the *LoG* and the Sauvola binarizations, some compromised borders (as can be seen on Fig. 3 with the *Hymenolepis diminuta*) can be detected with their correct morphologic characteristics, accurately classified and verified (Fig. 5).

The next step was the Watershed algorithm, which is used to separate objects that are in contact within an image (2f). This method treats pixel values as a local elevation to later flood the basins from pre-defined markers, until basins attributed to different markers meet on watershed lines.

Finally, the process of morphological filtering eliminates shapes that are outside a size threshold and those that are larger and thinner than the target objects. This is possible by applying the circularity shape metric and considering the relation between the major and minor axis lengths; this represents all objects that are outside the mean ± 2 standard deviations for the distribution of all eggs. All the objects that passed the form restrictions were labeled for their further characterization and classification.

### 2.3 Object characterization

After image processing, the system has a set of isolated tagged objects present in an image and a set of features for each of the tagged objects. These characteristics were selected because they allowed better description and differentiation between each of the different species of helminth eggs included in this system, as well as with the rest of the detritus.

The characteristics used for the morphological and gray level classifications can be separated into two groups: shape and pixel gray level.

• Shape characteristics include area, perimeter, circularity metric, major and minor axis, major and minor axis relation, and Hu first invariant moment.
• Pixel level characteristics are mean, standard deviation, kurtosis (a measure of the “peakedness” of the grayscale probability distribution), entropy, and object-background relation.

Subsequently, in order to improve the classification of the system and prevent overlapping between certain species that might be similar in size, shape, and even gray pixel level, an extra characteristic was calculated. This characteristic is a texture descriptor known as Local Binary Pattern (LBP) (Ojala et al., 2002). The LBP labels the pixels of an image by comparing the value of each pixel with its neighbors and considers the result as a binary chain number, which may thus be converted to decimal. That number is stored to create a histogram that defines the relationship that exists between the pixels of an image or a particular region.

To calculate the binary chain, the first step is to determine which pixels are going to be considered as neighbors of the central pixel. This is done by using a circular neighborhood denoted by the number of sampling points and the radius. (P,R).

The sampling point coordinates $\left(x_{p},\;y_{p}\right)$ are calculated using the expression:

(3) $$x_{p}=x_{c}+Rcos\left(\frac{2\pi p}{P}\right),y_{p}=y_{c}-Rsin\left(\frac{2\pi p}{P}\right)$$

where:

$x_{c}$ and $y_{c}$ are the central pixel where the LBP is going to be calculated.

P represents the number of sampling points.

R represents the radius of the neighborhood.

When the sampling coordinates do not fall at integer positions, intensity values are linearly interpolated. After that, the neighboring pixel values are compared with the central pixel to generate the binary chain. The binary chain is converted to decimal and that number can be stored to create a histogram that defines the relationship that exists between the pixels of an image or a particular region. This method was useful to characterize the pixel level texture of each egg species.

(4) $$LBP_{P,R}\left(g_{c}\right)=\sum_{p=0}^{P-1}s\left(g_{p}-g_{c}\right)2^{P}$$

where.

gcIs the central pixel at $\left(x_{c}\;,\;y_{c}\right)$ coordinates.

$g_{p}\;|\;p=0,\ldots ,P-1$ are the values of the neighbors.

P represents the number of sampling points.

Eq. (4) represents a “texture unit” composed of P + 1 sampled element (central pixel included). There are 2^P^ possible texture units describing spatial patterns in a neighborhood of P points. In addition, $LBP_{P,R}$ achieves invariance against any monotonic transformation by considering the sign of the differences in$\;s\left(g_{p}-g_{c}\right)$. The comparison function s (x) is defined as:

(5) $$s\left(x\right)=\{\begin{array}{c} 1\;ifx\geq 0\\ 0ifx<0 \end{array}$$

**Fig. 6:**
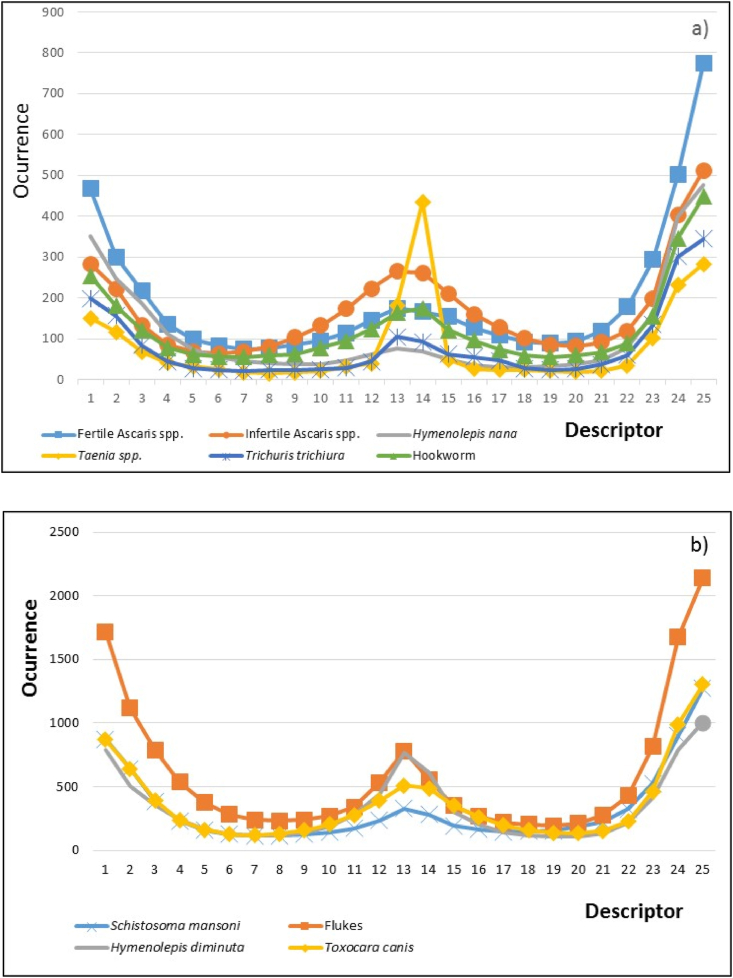
Gray level texture for each species of egg: **a)** Small egg species, **b)** Larger egg species.

This method was useful to characterize the pixel level texture of each egg species, as shown in Fig. 6.

Three K neighbors’ classifiers were used, based on a weighted Mahalanobis distance; two for local binary patterns (LBP), and one for morphological and gray level characteristics.

The Mahalanobis distance $D_{M}$of an observation $x={\left(x_{1},x_{2},x_{3},\ldots \;,x_{N}\right)}^{T}$ from a group of with mean $\mu ={\left(\mu_{1},\mu_{2}\mu_{3},\;\ldots ,\mu_{N}\right)}^{T}$ and covariance matrix S is defined as:

(6) $$D_{M}\left(x\right)=\sqrt{{\left(x-\mu \right)}^{T}S^{-1}\left(x-\mu \right)}$$

To determine the class of an object, the result was calculated by adding all the scores of the three K neighbor classifiers, and the class with more votes was chosen. Each of the training data consists of a set of vectors and the class labels associated with them; also, a single number “k" was given. This number decides how many neighbors influence the classification. In this case, five neighbors for the classical characteristics vector, and three for the 4 and 8 distances LBPs were used. This was useful because it gives an additional vote to correctly classify the possible egg, considering not only the form and gray level characteristics but also the texture. To confirm the result, a statistical verification process was applied; if three of the morphological and gray level characteristics of the object were outside the mean ± 2 standard deviations, the egg classification was considered unreliable, and the object class was defined as “not an egg”. In this step, segmented objects with characteristics that were never found present in an actual egg are eliminated to improve specificity; these special characteristics are usually outside the mean ± 3 standard deviations.

**Fig. 7:**
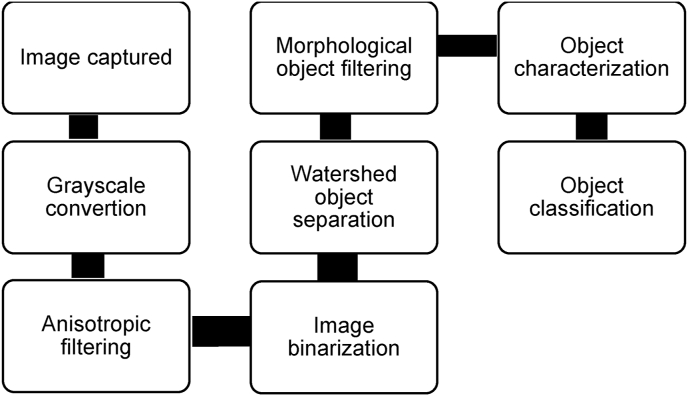
Image processing sequence.

In summary, the image processing path consists of a series of steps (Fig. 7). The main steps focused on transforming the image to identify certain characteristics of the detected objects and compared them with morphological parameters characteristic of selected helminth eggs.

The parameters used to evaluate the proficiency of the system were:

*Sensitivity (Se):* corresponds to the percentage of true positives in relation to the total number of counted eggs:

(7) $$Se=\;\frac{Tp}{\left(Tp+Fn\right)}$$

where.

Tp is the number of true positives.

Fn is the number of false negatives.

“Tp” was regarded as the number of helminth eggs correctly identified and “Fn” as the number of existent helminth eggs that were not correctly identified in the sample.

*Specificity (Sp)* is the percentage of true negatives in relation to all the objects detected excluding actual eggs:

(8) $$Sp=\frac{Tn}{\left(Tn+Fp\right)}$$

where.

Tn is the number of true negatives.

Fp is the number of false positives.

“Tn” was regarded as the number of objects other than helminth eggs that were correctly identified as “other objects”, and “Fp” as the number of objects other than helminth eggs that were mistakenly identified as eggs.

## 3 Results and discussion

### 3.1 Image scaling

During the first stage of software validation, the tests performed in international laboratories in Brazil, Colombia and France (Table 1) did not work as expected. This was a result of the use of different designs for the microscope components used to acquire the images and different microscope configurations (different focal distances). As a result, a new software module was therefore developed to automatically scale/reduce the images and allow the software to process them. This modification resulted in some limitations in terms of image details that reduced the accuracy of the results when the image scale required data interpolation (pixel information was lost and eggs were not detected).

**Table 1:** List of the selected laboratories.

Contact	Address
Dr. Sandrine Banas	UMR 7564, LCPME, Faculté de Pharmacie, 5 rue Albert Lebrun BP 80403 54001 Nancy Cédex, France.
Dr. Claudia Campos	Department of Microbiology, Departamento de Microbiología, Pontificia Universidad Javeriana, Bogotá-Colombia.
Dr. Maria Inês Zanoli Sato	Departamento de Análises Ambientais, EL CETESB-, Companhia Ambiental, São Paulo- SP, Brasil.
Dr. Maria Tereza Pepe Razzolini	Departamento de Saúde Ambiental, Faculdade de Saúde Pública/USP, São Paulo-SP, Brasil.

To solve this problem, it was necessary to find a method capable of addressing it while preserving form, size, and texture. This was possible by employing a Gaussian pyramid (Adelson et al., 1984). In order to calculate the relationship between μm and pixel size, an image of a microscope calibration slide/stage micrometer taken with the same microscope-camera setup as that used to create the image of the sample is required. This provides information to the software about the actual size of the objects and, subsequently, the system can rescale an image to match the size of the images used for calibration. This scaling method allows the system to analyze images of any size (within a certain range) while maintaining the characteristics of the objects (size, color, and texture) largely unaltered.

Images of environmental samples, including untreated and treated wastewater, sludge, biosolids, soil and excreta were acquired locally and from international environmental laboratories. Application of the HEAD system showed average sensitivity and specificity for validation of 94% and 97%, respectively. In total, these images contained more than 400 helminth eggs of different species and the software counted approximately 3000 objects (artifacts) present in the images. This represents a considerable improvement on the results obtained using the previous version of the software as reported by Jiménez et al. (2016). The benefits of the software in terms of cost reduction and time saved for the analysis of various types of environmental samples were evident, and it provided a useful technique for counting eggs of different species of helminth.

These results show greater sensitivity than those reported by Yang et al. (2001) where an 86% average was recorded. In contrast, this sensitivity is below that obtained by Avci and Varol (2009). However, in that study the classification ratio (sensitivity) of 98% was achieved with 2400 eggs in the validation group, showing the importance of increasing the number of training and validation egg images. This occurs because the chance of achieving an improved description of the eggs’ form and texture characteristics increases with a high number of examples, lowering the probability of classification or verification errors due to a strong description.

### 3.2 Development of the HEAD software application online

The new Software version in C# with. NET framework has been assigned to a remote server to establish a server-client web service model. This allows to images to be received and processed automatically, as well as direct reporting of the results without the need for trained operators. For this, a cloud computing service was proposed, because it automatically adapts to demand-based services, allows a payment-for-use scheme, represents the perfect tool for our server-client web service model, and provides a flexible tool with multiple security protocols and easy access. It is expected that the software developed will significantly reduce the costs necessary to detect and quantify helminth eggs with a high level of accuracy. This represents a tool not only for microbiologists and researchers but also for various agencies involved in sanitation, such as environmental regulation agencies, which currently require highly-trained technicians. In addition, the simplicity of the device contributes to control of the contamination of wastewater, and also sludge, biosolids, soils, and excreta samples, even in poor and isolated communities.

The migration to the new cloud-hosting provider will benefit both the development and the maintenance of the HEAD solution over the long term. This will also allow the augmentation of the solution's capabilities in the future. As part of this migration process, a new staging environment was created for reviewing the developers' work on wiring the User Interface (UI) and Application Programming Interface (API) for the web application, although there is still active development occurring within it. The developers will be continuing with the wiring for the web application. This will support users of the web application who may be using a mobile device such as a phone or tablet.

**Fig. 8:**
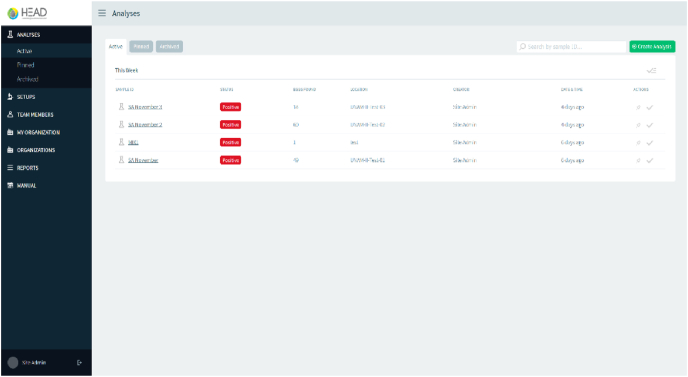
HEAD home page.

As shown in Fig. 8, the HEAD website has different options to perform the analysis. In the side menu the user can find the “Analysis” section in which a new test can be created, in the “Setup” section the user can update the system configuration data with the microscope camera and scale image information. In the “Team members, My Organization and Organizations” section, the user can edit the organization details. In the “Reports” section an excel file can be created with all the details of the test performed, while in the final section, “Manual”, the user can find the general instructions for the online system.

**Fig. 9:**
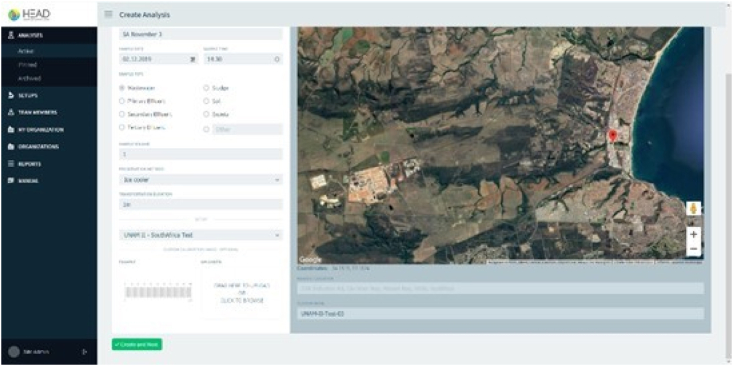
Sample identification.

The analysis process on the web page consists of the information of the sample (date and sample time, type, volume, preservation method, transportation duration and sampling site location). It allows adjustment of the scale pixel size, and the attachment of an image with a stage micrometric (Fig. 9).

**Fig. 10:**
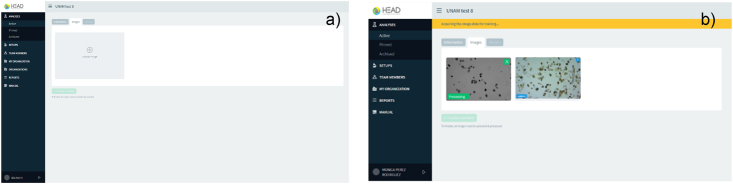
a) Image upload **b)** Image procesed and analyzed.

Once the user has processed the sample and has acquired potential images of helminth eggs of sufficient quality (illumination, contrast and sharpness), they may proceed to upload them as shown in Fig. 10a. If the image is correctly accepted, it will show the label “processing” and finally “analyzed” (Fig. 10b).

**Fig. 11:**
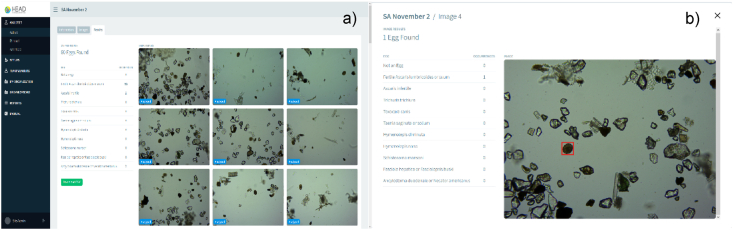
a) General results of the set of images, **b)** Identification in each image. Helminth egg image database Server-client web service.

Fig. 11 shows the results of helminth egg analysis, Fig. 11a shows the identification and quantification of the species found in the complete set of analyzed images and Fig. 11b shows the location of every species of helminth egg per image included in the software.

**Fig. 12:**
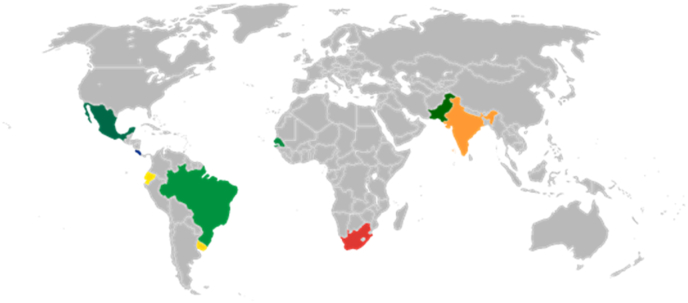
Laboratories selected in nine different countries: Mexico, Costa Rica, Ecuador, Brazil, Uruguay, Senegal, South Africa, Pakistan, and India.

In order to establish the minimum resolution under which the system maintains acceptable specificity and sensitivity values and correct functioning on the web service, eleven laboratories from nine different countries were used. Selection was based on their geographical location, experience in the analysis of helminth eggs in environmental samples, potential contribution to diagnosis, and practice in controlling helminthiasis in developing countries. Each selected laboratory received a Carl Zeiss AxioLab A1 optical microscope and an Imaging Development Systems UI-1480LE-C-HQ USB2 color camera. Each laboratory was tasked to acquire and upload 50 high quality digital images of identified helminth eggs in different environmental samples processed at their laboratories each month over the course of one year. To collect homogenous images, all photographs were to be acquired using a 10× objective, under adequate illumination, with 2560 × 1920 pixel resolution without compression, and with adequate contrast and sharpness. Table 2 indicates the institutes or organizations that participated in this training and validation process, as well as their corresponding contacts (Fig. 12).

**Table 2:** List of the selected laboratories.

Contact	Address
Dr. Imran Hashmi	National University of Sciences and Technology (NUST), Sector H-12 NUST, Islamabad, Pakistan.
Ms. Kanza Naseer	
Dr. Srikanth Mutnuri,	Department of Biological Sciences, Birla Institute of Technology & Science, Pilani K K Birla Goa Campus, NH 17 B, Zuarinagar 403 726, Goa, India.
Dr. Radhamani V.	
Dr. Bipin G. Nair	Amrita School of Biotechnology, Amrita Vishwa Vidyapeetham, Amritapuri Camous, Clappana P·O., Kollam 690 525, Kerala, India.
Dr. Sanjay Pal	
Dr. Naveen Suresh	
Dr. Colleen Archer	Pollution Research Group, Discipline of Chemical Engineering, Howard College Campus, University of KwaZulu-Natal, Durban 4041, South Africa.
Dr. Danica Naidoo	
Dr. Kerry Lee Philp	
Dr. Seydou Niang	Laboratory of Wastewater Treatment and Water Pollution, University Cheikh Anta Diop, BP 206, Dakar, Senegal.
Dr. Ana María Acuña	Dpto. de Parasitología y Micología, Facultad de Medicina, Universidad de la República. Instituto de Higiene. Av. Alfredo Navarro 3051, CP 11600, Montevideo, Uruguay.
Dr. Anaydé Lena Lacuesta	
Fernanda Méndez Pignatta (Br.)	
Alejandra Valentín (Br.)	
Natalia Barboza (P·C)	Laboratorio Ambiental de la Dirección Nacional de Medio Ambiente, Ministerio de Vivienda Ordenamiento Territorial y Medio Ambiente, Avenida Italia 6201, Modulo 14, 1^er^ piso, C.P. 11500, Montevideo, Uruguay.
Gabriela Pistone (B Sc.)	
Ernesto Alfaro Arrieta (B Sc.)	Laboratorio Nacional de Aguas, Instituto Costarricense de Acueductos y Alcantarillados, Calle Central Contiguo Av. 9, C.P. 30301, Tres Ríos, La Unión, Cartago, Costa Rica.
Dr. Gustavo Rafael Collere Possetti	
Julio Cezar Rietow (M Sc.)	
Maira Ruggi (M Sc.)	Sanepar (Companhia de Saneamento do Paraná) - Centro de Tecnologias Sustentáveis (CETs)
Dr. Audrei Margarida Basso Osinski	Rua Engenheiro Antônio Batista Ribas, 151 - Tarumã, Curitiba - Paraná, Brasil. CEP: 82 800-050.
Dr. Cynthia Castro Corrêa Malaghini	
Rafaella Storrer Mendes dos Santos	
Isabella Storrer Mendes dos Santos	
Dr. Manuel Calvopiña	Escuela de Medicina, Universidad De Las Américas (UDLA),
Dr. Maura Rojas	Calle José Queri s/n. Sede Queri Bloque 5, 2° piso, C.P. 170515 Quito, Ecuador.
Dr. Juan Manuel Méndez Contreras	Tecnológico Nacional de México/Instituto Tecnológico de Orizaba, Av. Oriente 9 No. 852. Col. Emiliano Zapata. CP 94320. Orizaba, Veracruz, México.

Server-client web service TensorFlow (TF) object detection model.

The final improvement applied was to generate a TF model, an open source library for numerical computation and machine learning. TF is used for image classification and object detection as the applications are easier to develop. It simplifies construction, training and deployment of the models. It also allows the recognition, and localization of multiple objects within an image; this is the aim for the detection of helminth eggs in microscope images (Alom et al., 2018 *and* Coy et al., 2019).

Using the images of helminth eggs provided by the international laboratories from different environmental samples (wastewater, soil, biosolids, excreta, and sludge), 1188 images were used to label the eggs presented, with a confidence threshold value of 0.5. The group of images was divided into three sets: a) training 80%, b) validation 10% and c) testing 10%. The training set was used to acquire the egg characteristics for the detection, the validation set for parameter tuning and the testing set to evaluate the TF model. Sensitivity and specificity values of the TF model evaluation were 96.82% and 97.96%, respectively.

## 4 Conclusions

The improved HEAD System is able to efficiently identify and quantify helminth eggs in commonly acquired wastewater samples with consistent results, i.e. *Ascaris lumbricoides* -fertile or infertile-, *Toxocara canis*, *Trichuris trichiura*, *Taenia solium, Taenia saginata, Hymenolepis diminuta, Hymenolepis nana, Schistosoma mansoni*, *Ancylostoma duodenale* and *Necator americanus* (Hookworm), *Fasciola hepatica* and *Fasciolopsis buski* (flukes). The main advantage of using this software is that it does not require highly trained personnel (i.e. expert parasitologists). Additionally, results are obtained with consistent reliability, sensitivity, and specificity, allowing the comparison of data among countries/regions.

HEAD reduces processing time for each image by identifying the main bottlenecks that reduce processing speed within the code. Parallel processing may be implemented and optimized to further reduce processing time (10–30 s). Finally, the HEAD System will be operating as a cloud computing or client-server service, and will allow the use of this application in developing countries without the need for a data connection application.

The system is a novel approach for identifying and quantifying a large number of helminth eggs in various environmental samples, including wastewater, sludge, biosolids, excreta, and soil. It gives consistent results, and averages for sensitivity and specificity of 96.82 and 97.96, respectively, for the online service. Its application will significantly reduce the time and costs necessary to perform helminth egg analyses by avoiding the need for highly qualified technicians. It relies on a rapid and precise processing path.

Additionally, the free to use web service will allow enumeration of helminth eggs in developing countries, not only for microbiology specialists and researchers, but for many professionals involved in their control such those working in treatment facilities, academic institutions, research laboratories, and regulatory bodies.

The use of this service is expected to reduce identification costs and will provide an option to promptly and reliably detect helminth eggs within a much wider community. Moreover, the model demonstrated the following advantages:

The enhanced simplicity provided by the system will be relevant to improve water quality control in many communities in the near future. As a result, the system will provide a valuable tool to improve sanitation conditions and health standards worldwide.

The potential use of this system, not just for helminth ova analysis but also for other types of pathogenic organisms, demonstrates the software's capability and flexibility to perform similar analysis within the same system, which adds value to its development. The TF model must be improved by increasing the images used and with more training iterations to obtain higher sensitivity and specificity values.

## Author statement

B. Jiménez: Conceptualization, Funding acquisition, Supervision.

C. Maya: Validation, Formal analysis, Writing-Original, Draft preparation, Supervision, Funding acquisition, Writing, Review and editing, Visualization.

G. Velásquez: Software, Validation, Formal analysis, Data curation.

J.A Barrios: Supervision, Visualization.

M. Pérez: Validation, Formal analysis, Data curation.

A. Román: Project manager, Writing, Review and editing.
